# Pathologic approach to Neonatal cholestasis with a simple scoring system for biliary atresia

**DOI:** 10.1007/s00428-023-03704-5

**Published:** 2023-11-27

**Authors:** Khadiga M. Ali, Khaled R. Zalata, Tarik Barakat, Sherine M. Elzeiny

**Affiliations:** 1 Pathology Department, Faculty of Medicine, Mansoura University, Elgomhoria Street, Mansoura, Eldakahliya 35516 Egypt; 2 Gastroenterology and Hepatology Unit, Mansoura Faculty of Medicine, Mansoura Children Hospital, Mansoura, Egypt

**Keywords:** Biliary atresia, Liver biopsy, Diagnostic score, Pathology, Neonatal cholestasis

## Abstract

A liver biopsy is essential for the diagnostic workup of persistent neonatal cholestasis (NC). The differential diagnosis of NC is broad, including obstructive and non-obstructive causes. In addition, histologic features of certain disorders may be non-specific in the early course of the disease. To evaluate liver biopsies using a practical histopathologic approach for NC and to define a simple scoring system for biliary atresia (BA) for routine clinical practice. From June 2006 to December 2021, liver biopsy specimens from infants with persistent NC were examined by two independent pathologists. The cases diagnosed as BA were correlated with clinical, radiologic, and laboratory data to calculate the final score. Four hundred and fifty-nine cases were enrolled in the study. They had a mean age of 63.94 ± 20.62 days and were followed for a median time of 58 (1–191) months. They included 162 (35.3%) cases of BA. On multivariate analysis, portal edema, ductular proliferation, cholangiolitis, and bile duct/ductular plugs were the histopathologic predictors of BA. A liver biopsy did perform well with a 95.1% sensitivity, 91.6% specificity, 86% PPV, and 97.1% NPV. At a cutoff of 5 of the scoring system, diagnosis of BA could be done with a sensitivity of 95.1% and a specificity of 100%. We have shown detailed histopathologic features of BA with more depth to infants aged ≤ 6 weeks. We have developed a simple scoring system using a combination of liver biopsy with non-invasive methods to increase the diagnostic accuracy of BA.

**Supplementary Information:**

The online version contains supplementary material available at 10.1007/s00428-023-03704-5.

## Introduction

Neonatal cholestasis (NC) is described as an early-life defect in bile production or flow that causes the liver to retain biliary substances. Neonatal cholestasis affects about 1 in every 2500 live births [10]. Biliary atresia (BA), which has been reported to occur in 35–41% of cases, is the most common condition to be the primary cause of NC, followed by metabolic disorders (9–17%), progressive familial intrahepatic cholestasis (PFIC) (10%), Alagille syndrome (2–6%), infectious diseases (1–9%), and other causes, including idiopathic cases (13–30%) [12].

It is critical to quickly identify the underlying cause in order to start the right surgical or medicinal treatment. Early surgical referral is necessary in BA to increase success rates. In the case of treatable metabolic diseases, a prompt diagnosis justifies prompt, targeted therapy and a better prognosis [19].

Infants with cholestatic jaundice require a liver biopsy as part of their diagnostic work-up. Interpretation of the biopsies in this clinical setting is challenging. The differential diagnosis of NC is broad, including obstructive and non-obstructive causes. In addition, histologic features of certain disorders may change over time or may be non-specific in the early course of the disease [23].

In this study, we provided a practical histopathologic assessment of liver biopsies performed in infants presented with persistent NC. Based on the data of patients with confirmed diagnoses, we suggested a simple diagnostic scoring system for BA for everyday clinical practice.

## Materials and methods

### Patient selection

A retrospective study was conducted from June 2006 to December 2021 to review the medical charts of patients referred to a tertiary care center at the pediatric gastroenterology and hepatology unit at Mansoura University Children’s Hospital (MUCH), Egypt, who underwent a liver biopsy as a part of the diagnostic workup for persistent NC. Infants aged less than 120 days old were enrolled in the study. Premature infants were excluded. Informed consent from the participant’s parents or guardians was obtained. Approval for this study was obtained from the Mansoura Faculty of Medicine institutional review board (R.21.11.1527).

### Histopathologic evaluation of liver biopsies

Formalin-fixed, paraffin-embedded blocks of liver biopsy samples were retrieved from the pathology archive of the pathology laboratory at MUCH. The initial evaluation was performed using hematoxylin–eosin (H&E), Masson trichrome, Periodic Acid Schiff (PAS) before and after diastase, and Prussian blue stains. Immunohistochemical (IHC) staining for CK7 was done before a diagnosis of the paucity of bile ducts was made and to highlight ductal proliferation characterized by having a patent lumen compared to ductular proliferation that appears as strings of cuboidal cells in a ductular configuration located outside the limiting plate in questionable cases.

All biopsy specimens contained at least 10 portal tracts [8]. The paucity of bile ducts was defined as a bile duct to portal tract ratio of less than 0.5 (normal 0.9–1.8) after performing several serial sections with an IHC-stained slide for CK7 [17].

**Fig. 1:**
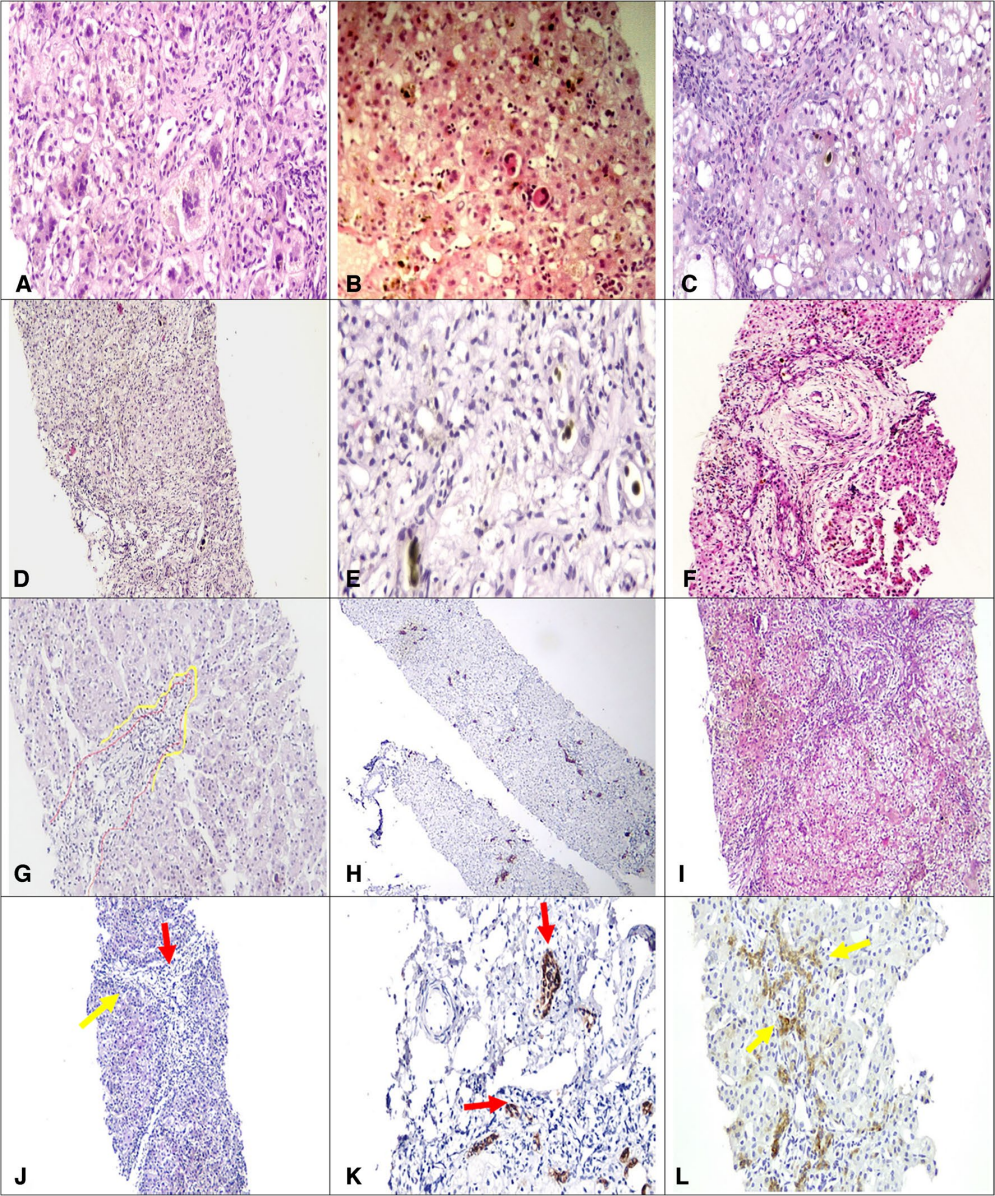
Representative images of the assessed histopathologic items. A 46-day-old girl with a neonatal hepatitis pattern showed mild cytoplasmic cholestasis with multinucleated giant hepatocytes (**A** H&E, magnification ×400). Another liver biopsy from a girl aged 39 days diagnosed with neonatal hepatitis pattern showed evident lobular extramedullary hematopoiesis (**B** H&E, magnification ×200). A 69-day-old boy was diagnosed with galactosemia and his liver biopsy showed moderate steatosis with cholestasis (**C** H&E, magnification ×200). A liver biopsy from a 45-day-old boy showed edematous portal tracts (**D** H&E, magnification ×100), where edema fluid dissects portal collagen around bile duct containing bile plugs (**E** H&E, magnification ×400) and bile ductules encircling a vessel resulting in ductal plate-like malformation (**F** H&E, magnification ×100). Bile ductular proliferation was assessed as mild focal “yellow line highlights ductular proliferation in < 50% of portal tract circumference outlined with red” (**G** H&E, magnification ×100), mild diffuse in most portal tracts (**H** IHC, CK7, magnification ×40) or moderate to marked (**I** H&E, magnification ×100). Both ductal proliferation and ductular proliferation (**J** H&E, magnification ×100) can be highlighted with IHC for CK7 where ductal proliferations have patent lumen “red arrow” compared to ductular proliferation forming strings of cuboidal cells in a ductular configuration located outside the limiting plate “yellow arrows” (**K, L** IHC, CK7, magnification ×400 and ×200 respectively)

Histopathologic evaluation of all liver biopsies was performed by two pathologists experienced in pediatric liver diseases who were given only the age of patients but were blinded to the clinical data or final diagnosis. They set together and agreed on the histopathologic features assessed. Fourteen histopathologic items were assessed (Fig. 1, Table 1). Cytoplasmic cholestasis was graded into mild (some but not all hepatocytes are stained with bile pigment), moderate (diffuse bile-stained hepatocytes sparing Kupffer cells), and marked (diffuse bile-stained hepatocytes and Kupffer cells). Bile ductular proliferation was defined as increased bile ductules at the interface and graded as absent, mild (involving less than half the circumference of the portal tract), either focal (involving < 50% of the portal tract) or diffuse (> 50% of portal tracts) pattern, moderate or marked when the proliferation exceeds half the circumference of the portal tract either in some or most portal tracts respectively [20] (Fig. 1).

**Table 1:** Demographic, clinical, and laboratory and histopathologic features of 459 infants with neonatal cholestasis

Features	*N* (%)	Features	*N* (%)
Age groups		Sex	
28–60 Days	200 (43.6%)	Boys	276 (60.1%)
61–90 Days	213 (46.4%)	Girls	183 (39.9%)
91–120 Days	46 (10%)		
Clay stool		Hepatomegaly	
Present	162 (35.3%)	Present	202 (44%)
Diarrhea/failure to thrive		Congenital anomalies	
Present	19 (4.1%)	Present	71 (15.5%)
TORCH screening^a^			
Positive	64 (13.9%)		
GGT^b^ (IU/L) in different age groups		Radiology	
	median (IQR^**c**^)	Suggestive of BA	134 (70.1%)
28–60 days	281 (89–509)	Not BA	322 (29.2%)
61–90 days	302 (97.5–597)	Non conclusive	3 (0.7%)
91–120 days	164 (41.5–630.25)		
Histopathologic features			
**1.** Portal fibrosis		2. Bile ductular proliferation	
Absent	95 (20.7%)	No	161 (35.1%)
Mild fibrosis	170 (37%)	Mild focal	76 (16.6%)
Moderate fibrosis	117 (25.5%)	Mild diffuse	77 (16.8%)
Advanced fibrosis	76 (16.6%)	Moderate or marked	145 (31.5%)
Cirrhosis	1 (0.2%)		
3. Portal tract edema		4. Ductal plate malformation	
Present	119 (25.9%)	Present	47 (10.2%)
5. Cholangiolitis		6. Bile duct/ductular plugs	
Present	102 (22.2%)	Present	250 (54.5%)
7. Portal inflammation		8. Parenchymal inflammation	
No	3 (0.7%)	No	241 (52.5%)
Mild	353 (76.9%)	Mild	192 (41.8%)
Moderate or marked	103 (22.4%)	Moderate or Marked	26 (5.7%)
9. Paucity of intrahepatic bile ducts		10. Secondary siderosis	
Present	78 (17%)	Present	136 (29.6%)
11. Extramedullary hematopoiesis		12. Giant-cell transformation	
Present	196 (42.7%)	Present	182 (39.7%)
13. Cholestasis		14. Steatosis	
Mild	120 (26.1%)	Absent	419 (91.3%)
Moderate	335 (73%)	Mild<33%	7 (1.5%)
Marked	4 (0.9%)	Moderate 33%-66%	24 (5.2%)
		Marked >66%	9 (2%)
Follow up result		Cause of death (96 cases)	
Living/dead	363 (79.1%)/ 96 (20.9%)	Pneumonia	7 (7.3%)
		Sepsis	13 (13.5%)
		Hepatic encephalopathy	76 (79.2%)
Diagnosis pathologically	*N* (%)	Final clinical diagnosis	*N* (%)
BA^**d**^	179 (39%)	BA	162 (35.3%)
Paucity	78 (17%)	Paucity and alagille	83 (18.1%)
NH^**e**^	143 (31.2%)	NH	116 (25.3%)
Cholestasis with steatosis	40 (8.7%)	PFIC 3^**6**^	12 (2.6%)
Intrahepatic cholestasis	19 (4.1%)	Congenital CMV infection	2 (0.4%)
		Choledocal cyst	4 (0.8%)
		PFIC-1 and PFIC-2^**f**^	18 (3.9%)
		Metabolic	54 (11.8%)
		Free	5 (1.1%)
		Others	3 (0.7%)

^**a**^*TORCH* toxoplasmosis, rubella cytomegalovirus, and herpes simplex, ^**b**^*GGT* gamma-glutamyl-transferase, ^**c**^*IQR* inter-quartile ratio, ^**d**^*BA* biliary atresia, ^**e**^*NH* neonatal hepatitis, ^**f**^*PFIC* progressive familial intrahepatic cholestasis

Steatosis was graded into 4 grades, absent (< 5%), mild (5–33%), moderate (34–66%), or marked (≥ 67%) [15]. Other features are assessed either present or absent as giant cell transformation of hepatocytes, extramedullary hematopoiesis (EMH), portal edema which is characterized by portal tract expansion with clearing, ductal plate malformation (DPM) where the bile ducts are arranged around the central fibrovascular core and bile duct/ductular plugs. Fibrosis was assessed as absent, mild fibrosis (expansion of portal tracts without bridging), moderate fibrosis (presence of occasional bridging fibrosis), advanced fibrosis (presence of marked bridging fibrosis with occasional nodules), and finally development of cirrhosis.

When features of obstructive cholestasis were present as portal edema, expansion by fibrosis, bile ductular proliferation, and bile duct/ductular plugs with or without cholangiolitis), a suggestion of BA was made. The presence of hepatocyte disarray with giant cell transformation, EMH, and cholestasis with intact interlobular bile ducts was considered a neonatal hepatitis pattern of injury [6]. Pure intrahepatic cholestasis is characterized by the presence of bile inside the hepatocytes and/or bile canaliculi without significant portal or parenchymal alterations [17]. When steatosis is easily identified in addition to cholestasis, screening for metabolic disorders such as galactosemia, tyrosinemia, or hereditary fructose intolerance is suggested in the differential diagnosis [7]. When there is discordance between the two pathologists, they evaluate the discordant cases together to reach a final classification.

### Data collection

Age at liver biopsy, sex, laboratory findings (total and direct bilirubin, gamma-glutamyl-transferase (GGT), and TORCH (toxoplasmosis, rubella cytomegalovirus (CMV), and herpes simplex) serological test results were gathered. Additional tests, such as the assessment of alpha-1 antitrypsin levels, the study of metabolic inborn errors, and the analysis of the polymerase chain reaction for CMV in the blood were collected.

Radiologic evaluations of the biliary tree, any concurrent congenital anomalies, and the date of Kasai surgery, if performed, were collected from the electronic registry system. Clinical follow-up information was obtained. A scoring system formed of 4 main parameters with a total score of 7 was applied to all cases in an attempt to differentiate between BA and non-BA cases.

### Statistical analysis

For continuous variables, descriptive data were summarized as the mean ± standard deviation (SD) or median and quartiles, and for categorical variables, as numbers and percentages. Pearson correlation coefficients were calculated for pairs of histologic features to characterize their associations. To evaluate the association between histologic characteristics and BA, univariate and multivariate logistic regression were performed. Sensitivity, specificity, and positive and negative predictive (PPV, NPV) values were calculated with histologic assignment as the test condition, and the clinical diagnosis (BA or non-BA) was considered as the genuine illness state of each patient. The receiver operating characteristic (ROC) curve’s area under the curve (AUC) was used to evaluate the diagnostic performance of each variable and score. Statistical analysis was performed by using the Statistical Package for Social Science (SPSS) version 22 (Armonk, NY: IBM Corp).

## Results

Four hundred and fifty-nine children were enrolled in the study. They included 276 boys and 183 girls. They had a mean age of 63.94 ± 20.62 days and were followed for a median time of 58 (1–191) months. Table 1 demonstrates the clinical, laboratory, and histologic features of all cases.

### Clinically confirmed BA versus non-BA cases

We documented 162 (35.3%) cases of BA out of all 459 enrolled cases (Table 2). It was noted that with increasing patient age, the GGT level increased in the BA group (from 474 to 754 IU/L) compared to the decrease in the non-BA group (from 121 to 67 IU/L).

**Table 2:** Demographic, clinical, laboratory, and histopathologic features of BA and non-BA groups

	Non-BA^a^ group *N* = 297	BA^a^ group *N* = 162	*P*-value*
Age groups			
28–60 Days	132 (44.4%)	68 (42%)	*P* = .74
61–90 Days	134 (45.1%)	79 (48.8%)	
91–120 Days	31 (10.4%)	15 (9.3%)	
Sex			
Male	187 (63%)	89 (54.9%)	*P* = .09
Female	110 (37%)	73 (45.1%)	
Clay stool: Present	0	162 (100%)	*P* < .001
Diarrhea/failure to thrive: Present	19 (6.4%)	0	*P* < .001
Hepatomegally: Present	90 (30.3%)	112 (69.1%)	*P* < .001
Congenital anomalies: Present	62 (20.9%)	9 (5.6%)	*P* < .001
GGT^b^ (IU/L) in different age groups	Median (IQR^**c**^)		
28–60 Days	121 (57-359.75)	474 (360.5-613.25)	*P* < .001
61–90 Days	124.5 (62-281.75)	567 (370-781)	*P* < .001
91–120 Days	67 (35-169)	754 (430-891)	*P* < .001
TORCH^d^ screening: Present	36 (12.1%)	28 (17.3%)	*P* = .13
Radiology			
Intact	296 (99.7%)	26 (16%)	*P* < .001
Suggestive of BA	0	134 (82.7%)	
Non-conclusive	1 (0.3%)	2 (1.2%)	
Histopathologic features			
1. Portal fibrosis			
Absent	94 (31.6%)	1 (0.6%)	*P* < .001
Mild fibrosis	138 (46.5%)	32 (19.8%)	
Moderate fibrosis	43 (14.5%)	74 (45.7%)	
Advanced fibrosis	22 (7.4%)	54 (33.4%)	
Cirrhosis	0	1 (0.6%)	
2. Portal tract edema: Present	3 (1.0%)	116 (71.6%)	*P* < .001
3. Bile ductular proliferation			
No	159 (53.5%)	2 (1.2%)	*P* < .001
Mild focal	71 (23.9%)	5 (3.1%)	
Mild diffuse	33 (11.1%)	44 (27.2%)	
Moderate or marked	33 (11.4%)	111 (68.5%)	
4. Ductal plate malformation			*P* < .001
Present	8 (2.7%)	39 (24.1%)	
5. Bile duct/ductular plugs	91 (30.6%)	159 (98.1%)	*P* < .001
Present			
6. Portal inflammation			*P* = .01
No	3 (1.0)	0	
Mild	215 (73.1%)	138 (85.2%)	
Moderate or Marked	79 (26.9%)	24 (14.8%)	
7. Cholangiolitis: Present	35(11.8%)	67(41.4%)	*P* < .001
8. Paucity of intrahepatic bile ducts			*P* < .001
Present	77 (25.9%)	1 (0.6%)	
9. Secondary siderosis			*P* = .09
Present	80 (26.9%)	56 (34.6%)	
10. Cholestasis			*P* < .001
Mild	104 (35%)	16 (9.9%)	
Moderate	192 (64.6%)	143 (88.3%)	
Marked	1 (0.3%)	3 (1.9%)	
11. Parenchymal inflammation			*P* < .001
No	116 (39.1%)	125 (77.2%)	
Mild,	156 (52.5%)	36 (22.2%)	
Moderate or marked	25 (8.4%)	1 (0.6%)	
12. EMH^e^			*P* < .001
Present	154 (51.9%)	42 (25.9%)	
13. Giant-cell transformation			*P* < .001
Present	144 (48.5%)	38 (23.5%)	
14. Steatosis			*P* < .001
Absent	257 (86.5%)	162(100%)	
Present	40 (13.5%)	0	
Follow up result			
Dead	45 (15.2%)	51 (31.5%)	*P* < .001
Living	252 (84.8%)	111 (68.5%)	
Cause of death (96 cases)			
Hepatic encephalopathy	29 (64.4%)	47 (92.2%)	*P* = .003
Pneumonia	5 (11.1%)	2 (3.9%)	
Sepsis	11 (24.4%)	2 (3.9%)	
Follow up (in months)	75 (22-120)	38 (6–75.25)	*P* < .001
Median (IQR^**3**^)			

^**a**^*BA*, biliary atresia; ^**b**^*GGT*, gamma-glutamyl-transferase; ^**c**^*IQR*, inter-quartile ratio); ^**d**^*TORCH*, toxoplasmosis, rubella cytomegalovirus, and herpes simplex); ^**e**^*EMH*, extramedullary hematopoiesis

^*^P < .05 is significant

At the histopathologic level, most of the BA cases showed bridging fibrosis (79.1 %) and moderate to marked bile ductular proliferation (68.5%) (*P* < .001). However, bile ductular proliferation was present to a variable degree in 46.5% of non-BA cases.

One hundred fifty-four cases out of 162 BA cases (95.1%) were clearly suggested on histopathologic examination, while the remaining 8 cases were missed as other diagnoses (7 cases with neonatal hepatitis pattern and one as paucity of intrahepatic bile duct). The mean age of the missing 8 cases was 29.75 (28–32) days. They were all presented with a clay stool with a median GGT level of 443 IU/L (IQR 301.75–643.25). On histopathologic examination, 50% showed mild portal fibrosis and mild diffuse bile ductular proliferation in 5 cases (62.5%). On the other hand, those diagnosed as obstructive cholestasis (179) by histopathologic examination included 154 cases confirmed to be BA, 3 cases of choledochal cyst, 10 cases of PFIC-3, 7 cases as Alagille syndrome (paucity), and 5 metabolic causes including alpha-1 antitrypsin deficiency. The non-BA cases misdiagnosed histologically as obstructive cholestasis did not experience any unnecessary surgical intervention.

#### Kasai procedure

At a mean age of 75.46 months, 142 BA group cases (87.7%) underwent Kasai surgery. An intraoperative cholangiogram was performed for all patients. The excised atretic segment was received and labelled, and the diameter of the bile ductule was measured using a DP50 camera (Olympus, Tokyo, Japan) equipped with an Olympus BX31 microscope. Only 28 operated cases (19.7%) developed hepatic encephalopathy. Those who did not have a Kasai procedure (20 cases) included 11 cases presented late with advanced fibrosis, as well as 9 cases where the parents refused to have surgery due to their belief that natural herbs would solve the problem.

Because the patient’s age is critical for the Kasai procedure [10], we divided all 162 clinically proven BA cases into two groups according to age (≤ 6 weeks and > 6 weeks, Supp Table 5). Histopathologic features of obstruction such as portal edema, advanced portal fibrosis, and bile ductular proliferation were more common in infants > 6 weeks of age (*P* < .001).

#### Concordance of liver biopsy with final diagnosis

When studying the histopathologic concordance with the clinical diagnosis of BA, a liver biopsy assessed using the strict histopathologic criteria (Table 1) did perform well, with a sensitivity of 95.1%, specificity of 91.6%, PPV of 86%, and NPV of 97.1%. Concordance between the histopathologic and the clinical diagnosis of BA was 0.846 (perfect agreement).

### Univariate and multivariate analysis of features associated with BA

The histopathologic predictors of BA identified by univariate analysis (Table 3) included portal edema, moderate and advanced portal fibrosis, ductular proliferation (mild diffuse or moderate/marked), bile duct/ductular plugs, DPM-like pattern, and cholangiolitis in addition to the absence of EMH or giant cell transformation of hepatocytes. Only portal edema, ductular proliferation (mild diffuse or moderate/marked), bile duct/ductular plugs, and cholangiolitis are the histopathologic predictors of BA according to multivariate analysis (Table 3).

**Table 3:** Univariate and multivariate analysis of histopathologic features associated with biliary atresia

Parameter	Univariate analysis		Multivariate analysis	
	HR ^a^(95% CI^b^)	*P* value*	HR ^a^ (95% CI^b^)	*P* value*
1. Portal tract edema	247.13 (75.37–810.32)	*P* < .001	87.423 (22.22–343.97)	*P* < .001
2. Ductular proliferation				
Mild diffuse	106 (24.47–459.09)	*P* < .001	91.82 (11.68–721.71)	*P* <. 001
Moderate or marked	228.24 (53.35–976.20)	*P* < .001	177.20 (21.88–1435.34)	*P* < .001
3. Bile duct/ductular plugs	119.98 (37.29-386.01)	*P* < .001	26.599 (6.54-108.21)	*P* < .001
4. Ductal plate malformation	11.45 (5.2-25.22)	*P* < .001	0.679 (0.178-2.6)	*P* < .001
5. Portal fibrosis				
Moderate fibrosis	7.89 (4.83–12.89)	*P* < .001	1.37 (0.701–2.69)	*P =* .35
Advanced fibrosis	23.55 (4.97–111.49)	*P* < .001	7.91 (0.687–90.98)	*P =* .10
6. Cholestasis				
Moderate	4.81 (2.74–8.55)	*P* < .001	1.01 (0.438–2.34)	*P =* .98
Marked	19.5 (1.91–199.14)	*P* < .001	0.685 (0.043–10.86)	*P =* .79
7. Cholangiolitis	5.28 (3.29–8.6)	*P* < .001	3.24 (1.86–7.24)	*P =* 0.03
8. Giant-cell transformation	0.326 (0.212–0.500)	*P* < .001	0.711 (0.301–1.68)	*P =* .44
9. EMH^c^	0.310 (0.204–0.473)	*P* < .001	0.807 (0.345–1.88)	*P =* .62

Abbreviations: ^**a**^*HR* hazard ratio, ^**b**^*CI* confidence interval, ^**c**^*EMH* extramedullary hematopoiesis

*P < .05 is significant

### Design of the diagnostic score for BA

There is no single diagnostic procedure that appears to be clearly superior for diagnosis of BA, rather a few diagnostic tools are used collectively to reach an early and accurate diagnosis. BA can only be correctly diagnosed by correlating the clinico-radiological features with histological findings [13]. In this study, all patients underwent an abdominal ultrasound, which gave a detailed report about the gall bladder length, contractility, triangular cord sign, and extra and intrahepatic biliary radicles with a clear comment on any abnormality as if the gallbladder (non-contractile or rudimentary or non-visualized). When radiology was non-conclusive or negative and the liver biopsy showed a picture of obstructive cholestasis, hepatobiliary scans (HIDA showing no excretion of radiotracer) and intraoperative cholangiogram were done to reach a definite diagnosis.

Serum levels of GGT are not required, but their high elevation (10 times normal) supports the diagnosis. Patient age was taken into consideration when assessing both the liver biopsy pathologically and the level of GGT. The AUC of serum GGT levels in relation to age group was 0.812 in neonate ≤ 60 days, 0.868 in infants aged 61–90 days, and 0.940 in those aged 91–120 days (Supp Figure 2). The cutoff of GGT in patients with BA ≤ 60 days was 309.5 IU/L with 82.4% sensitivity and 72.7% specificity. The cutoff increased to 347.5 IU/L with increasing age (61–90 days) with 88.6% sensitivity and 80.6% specificity. Despite that the cutoff value of GGT decreased to 295 IU/L at age (91–120 days), its sensitivity and specificity increased to 93.3% and 83.9%, respectively.

When all histopathologic features of obstruction are diffusely present (> 50% of portal tracts), a total score of 2 is given regardless of patient age. When some of these features are present or when all features are detected focally (< 50% of portal tracts), then referral to patient age is essential for scoring (Table 4).

**Table 4:** The diagnostic scoring system for biliary atresia

Items		Points
Clay stool	Present and persistent Absent	2
		0
Radiology	Suggestive of biliary atresia No abnormal features*	2
		0
Liver biopsy	Portal edema, bile duct/ductular plugs, and moderate to marked bile ductular proliferation (All^a^)	2
	Some but not all^b^	
	• ≤ 6weeks • >6 weeks (42 days) None	2 1 0
GGT^c^	Elevated (10 times normal for age )	1
	Normal	0
Total score		≥ 5 definite biliary atresia 4 probable ≤ 3 NOT biliary atresia

*No abnormal features after evaluating 2 different methods of assessment as ultrasound (US) and hepatobiliary scans

^a^All, indicates that these features should be present combined in more than 50% of portal tracts

^b^Some, either not all features present or present focally (<50% of portal tracts)

^c^*GGT* gamma-glutamyl-transferase

We assigned points to each of the 4 aforementioned parameters (Table 4). This score was then validated across all cases in the study (BA and non-BA cases). We found a sensitivity of 100% and a specificity of 93.3% at a cutoff of 3, 100% sensitivity and 100% specificity at a cutoff of 4, 95.1% sensitivity and 100% specificity at a cutoff of 5, and 78.4% sensitivity and 100% specificity at a cutoff of 6 points.

Since patient’s age can affect the histopathologic features of BA, we investigated all the study cases aged less than 6 weeks to evaluate how the diagnostic score will perform. Only 3 cases out of 19 cases of Paucity/Allagille and 2 cases of PFIC-3 got a score of 3, while 8 (30.8%) out of 26 cases of BA got a score of 4. When the total score is equivocal for BA, proceeding with an intraoperative cholangiogram may be the most effective and quick strategy to avoid the drawbacks of a delayed BA diagnosis, such as a delayed Kasai surgery and the higher possibility of a liver transplant.

## Discussion

A liver biopsy is advised for the purpose of diagnosing NC. Many cholestatic disorders are expressed differently over time. Additionally, liver biopsy can reveal disease-specific findings, such as the paucity of ducts in Alagille syndrome [2].

This study included a large number of liver biopsies in infants who presented with cholestasis. We investigated all histopathologic features in an attempt to clearly define those with obstructive versus non-obstructive cholestasis. Histopathologic characteristics closely related to BA (portal edema, bile ductular proliferation, and plugs) concurred with those made by Russo et al. [20, 21] and Zerbini et al. [25]. According to Ferry et al. [11], the primary characteristic of biopsies taken from BA patients is bile duct proliferation.

A small number of pathologists were involved in several published papers from single institutions on the reliability of liver biopsy for the diagnosis of BA [1, 10, 18]. In our study, the liver biopsy resulted in a concordance rate of 0.846 for the diagnosis of BA. Brough and Bernstein [3] found an accuracy of 93.7% for the pathologic diagnosis. Additionally, Ferry et al. [11] reported retrospectively that in 94% of 143 cases, the first liver biopsy accurately predicted the clinical diagnosis.

BA has been identified using GGT activity in serum. Serum GGT > 300 IU/L or a daily increase of 6 IU/L can be used to distinguish between BA and neonatal hepatitis. The accuracy of GGT measurement for the diagnosis of BA is increased by correlation with the reference range for GGT, which is dependent on age [5].

Many centers begin to rely on the serum level of matrix metalloproteinase-7 (MMP-7) for the diagnosis of BA. While the potential of MMP-7 to diagnose BA is clear, there is not much research performed on where exactly in the pathophysiology of BA, MMP-7 can be a factor. Sakaguchi et al. [22] and the review by Tang et al. [24] found that serum MMP-7 is a useful marker for the diagnosis of BA. However, Karbasian et al. [14] questioned the diagnostic value of MMP7 in differentiating BA from non-BA cases in the Middle Eastern population. They stated that MMP7 lacks enough specificity and lower diagnostic accuracy compared to GGT in diagnosing BA from other causes of NC. Our study is a retrospective one where serum MMP-7 was only introduced to our center by the year 2017. In addition, there is still debate about it together with the lack of clear cutoff values that distinguished infants with BA from those without [24].

Most of the scoring systems proposed for BA were based on histopathologic features to increase the accuracy of pathological evaluation of liver biopsy. Chen et al. [4] established a 21-point histological scoring system and found that a score of ≥ 8 had the best diagnostic utility to differentiate BA from other intrahepatic cholestasis histologically (sensitivity 94.7%, specificity 86.2%, accuracy 91.9%). Russo et al. [20] stated that the diagnostic accuracy of the needle biopsy was estimated to be 90.1%, whereas sensitivity and specificity for a diagnosis of BA are 88.4% and 92.7%, respectively. Zhao et al. [26] did not use the histopathologic feature in their scoring system and only relied on the use of glycochenodeoxycholic acid/chenodeoxycholic acid, clay stool, and GGT. A cutoff value of 15 from a total score of 41 identified BA with 90% sensitivity and 80% specificity in a validation cohort. Lee and Looi [16] used a detailed 15 points of 7 features assessed histopathologically only for the diagnosis of BA without including the clinical data. Their results showed that a score of ≥ 7 had the best diagnostic utility to differentiate BA from other intrahepatic cholestasis histologically (sensitivity 88%, specificity 94%, accuracy 92%). Our scoring system is similar to some extent to the scoring system for BA designed by El-Guindi et al. [9] who proposed a 12 points scoring system. A cutoff value of > 23.927 could discriminate BA from other causes of neonatal cholestasis with sensitivity of 100% and the accuracy was 98.83% in predicting BA. However, ours is simpler, easier to use in day-to-day practice, and included a larger study group (459 cases).

This study has several limitations. First, it is a single-center retrospective study. Second, this study did not address the prognostic value of histologic characteristics of a liver biopsy performed for NC. Third, this scoring system needs to be validated by other investigators.

In conclusion, we have shown detailed histopathologic features of BA with more depth to those presented earlier than 6 weeks of age and developed a simple scoring system for BA. However, it is impossible to make a diagnosis based solely on a biopsy but with correlation with clinical, radiologic, and laboratory information, the standard of care for these infants will take place.

### Supplementary information

ESM 1 (DOCX 40 kb)

ESM 2 (DOCX 23 kb)

## Abbreviations

BA: Biliary atresia
DPM: ductal plate malformation
EMH: Extramedullary hematopoiesis
NC: Neonatal cholestasis
PFIC: Progressive familial intrahepatic cholestasis

## Declarations

The authors confirm that the work complies with ethical standards.

## References

[1] Alagille D, Gautier M, Habib E, Dommergues J (1969). Pre-and postoperative hepatic biopsy data in prolonged cholestasis in infants. Study of 128 cases Arch Francaises de Pediatr.

[2] Bellomo-Brandao MA, Escanhoela CA, Meirelles LR, Porta G, Hessel G (2009). Analysis of the histologic features in the differential diagnosis of intrahepatic neonatal cholestasis. World J Gastroenterol.

[3] Brough AJ, Bernstein J (1974). Conjugated hyperbilirubinemia in early infancy: a reassessment of liver biopsy. Human Pathol.

[4] Chen G, Xue P, Zheng S, Chen L, Ma Y (2015). A pathological scoring system in the diagnosis and judgment of prognosis of biliary atresia. J Pediatr Surg.

[5] Chen X, Dong R, Shen Z, Yan W, Zheng S (2016). Value of gamma-glutamyl transpeptidase for diagnosis of biliary atresia by correlation with age. J Pediatr Gastroenterol Nutr.

[6] Cho S-J, Kim GE (2019) A practical approach to the pathology of neonatal cholestatic liver disease. Semin Diagn Pathol:375–388 10.1053/j.semdp.2019.07.004 31455583

[7] Cho S-J, Perito ER, Shafizadeh N, Kim GE (2021). Dialogs in the assessment of neonatal cholestatic liver disease. Human Pathol.

[8] Dezsofi A, Baumann U, Dhawan A, Durmaz O, Fischler B, Hadzic N, Hierro L, Lacaille F, McLin VA, Nobili V (2015). Liver biopsy in children: position paper of the ESPGHAN Hepatology Committee. J Pediatr Gastroenterol Nutr.

[9] El-Guindi MA-S, Sira MM, Sira AM, Salem TA-H, El-Abd OL, Konsowa HA-S, El-Azab DS, Allam AA-H (2014). Design and validation of a diagnostic score for biliary atresia. J Hepatol.

[10] Fawaz R, Baumann U, Ekong U, Fischler B, Hadzic N, Mack CL, McLin VA, Molleston JP, Neimark E, Ng VLJJopg, nutrition (2017). Guideline for the evaluation of cholestatic jaundice in infants: joint recommendations of the North American Society for Pediatric Gastroenterology, Hepatology, and Nutrition and the European Society for Pediatric Gastroenterology, Hepatology, and Nutrition. J Pediatr Gastroenterol Nutr.

[11] Ferry GD, Selby ML, Udall J, Finegold M, Nichols B (1985). Guide to early diagnosis of biliary obstruction in infancy: review of 143 cases. Clin Pediatr.

[12] Götze T, Blessing H, Grillhösl C, Gerner P, Hoerning A (2015). Neonatal cholestasis–differential diagnoses, current diagnostic procedures and treatment. Front Pediatr.

[13] Halder A, Patra S, Mandal B, Ray G, Ghosh R, Mukherjee S, Chatterjee U (2023) Differentiating biliary atresia from other causes of infantile cholestasis: an appraisal of the histomorphological changes on liver biopsy. Indian J Pathol Microbiol. Available from: https://www.ijpmonline.org/preprintarticle.asp?id=369085 10.4103/ijpm.ijpm_215_22 38084534

[14] Karbasian F, Mashhadiagha A, Anbardar MH, Ataollahi M, Dehghani SM, Honar N, Haghighat M, Imanieh MH, Sayadi M, Shahramian I, Aghsam A, Hosseini A, Mahadavi Mortazavi SM, Darban B, Avazpour A, Mirrahimi B, Ruzbahani AK, Tadayon A (2023). Questioning Diagnostic Value of Serum Matrix Metalloproteinase 7 for Biliary Atresia. J Clin Exp Hepatol..

[15] Kleiner DE, Brunt EM (2012) Nonalcoholic fatty liver disease: pathologic patterns and biopsy evaluation in clinical research. Semin Liver Dis 32:003–013. 10.1055/s-0032-1306421 10.1055/s-0032-1306421 22418883

[16] Lee WS, Looi LM (2009). Usefulness of a scoring system in the interpretation of histology in neonatal cholestasis. World J Gastroenterol WJG.

[17] López Panqueva RdP (2014). Approaches to pathological diagnosis of cholestatic diseases. Revista colombiana de Gastroenterología.

[18] Nezelof C (1972). Histological findings in neonatal hepatitis. Am J Dis Child.

[19] Nicastro E, Di Giorgio A, Marchetti D, Barboni C, Cereda A, Iascone M, D` Antiga L (2019). Diagnostic yield of an algorithm for neonatal and infantile cholestasis integrating next-generation sequencing. J Pediatr.

[20] Russo P, Magee JC, Anders RA, Bove KE, Chung C, Cummings OW, Finegold MJ, Finn LS, Kim GE, Lovell MA (2016). Key histopathological features of liver biopsies that distinguish biliary atresia from other causes of infantile cholestasis and their correlation with outcome: a multicenter study. Am J Surg Pathol.

[21] Russo P, Magee JC, Boitnott J, Bove KE, Raghunathan T, Finegold M, Haas J, Jaffe R, Kim GE, Magid M (2011). Design and validation of the biliary atresia research consortium histologic assessment system for cholestasis in infancy. Clin Gastroenterol Hepatol.

[22] Sakaguchi H, Ki K, Yasuda R, Sasaki H, Yoshimaru K, Tainaka T, Fukahori S, Sanada Y, Iwama I, Shoji H (2022). Serum matrix metalloproteinase-7 in biliary atresia: a Japanese multicenter study. Hepatol Res.

[23] Shneider BL, Brown MB, Haber B, Whitington PF, Schwarz K, Squires R, Bezerra J, Shepherd R, Rosenthal P, Hoofnagle JH (2006). A multicenter study of the outcome of biliary atresia in the United States, 1997 to 2000. J Pediatr.

[24] Tang X, Lv Y, Pu L, Ma J, Jin S, Xiang B (2022). Matrix metalloproteinase-7 as a diagnostic marker for biliary atresia: a systematic review and meta-analysis. Indian J Surg.

[25] Zerbini M, Gallucci S, Maezono R, Ueno C, Porta G, Maksoud J, Gayotto L (1997). Liver biopsy in neonatal cholestasis: a review on statistical grounds Modern Pathology: an Official Journal of the United States and Canadian Academy of Pathology. INC.

[26] Zhao D, Zhou K, Chen Y, Xie W, Zhang Y (2020) Development and validation of bile acid profile-based scoring system for identification of biliary atresia: a prospective study. BMC Pediatr 20:255. 10.1186/s12887-020-02169-8 10.1186/s12887-020-02169-8 PMC7251733 32460787

