# Supplementary material for: Pathologic approach to Neonatal cholestasis with a simple scoring system for biliary atresia
Source: Virchows Arch. 2023 Nov 27;484(1):93–102. doi: 10.1007/s00428-023-03704-5 (PMC10791702; doi:10.1007/s00428-023-03704-5)
Supplement: Supplementary file 1 — (DOCX 40 kb) [file 428_2023_3704_MOESM1_ESM.docx]

**A**

| 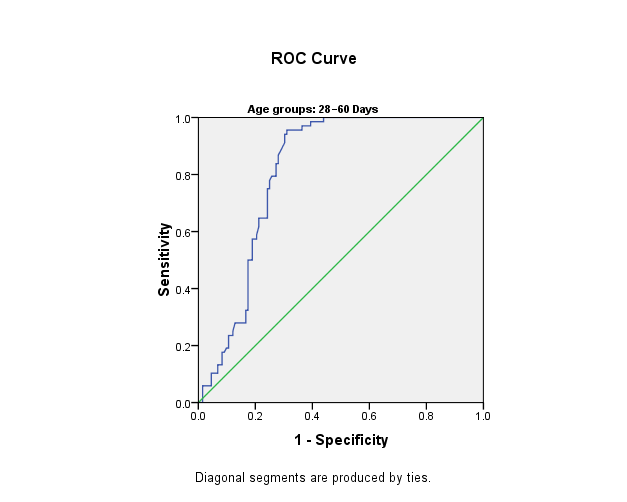 | 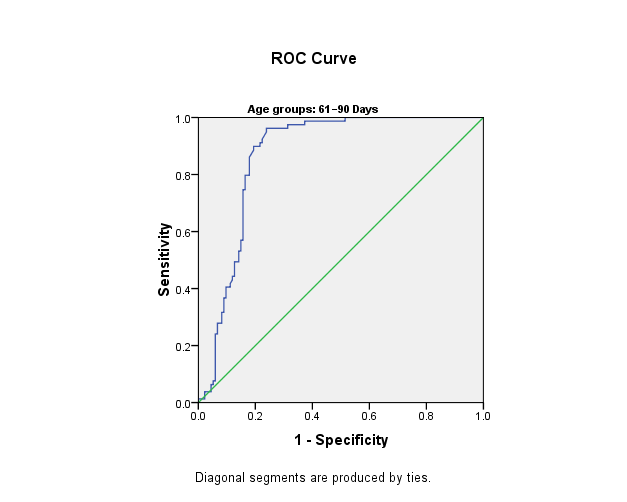  **A** | 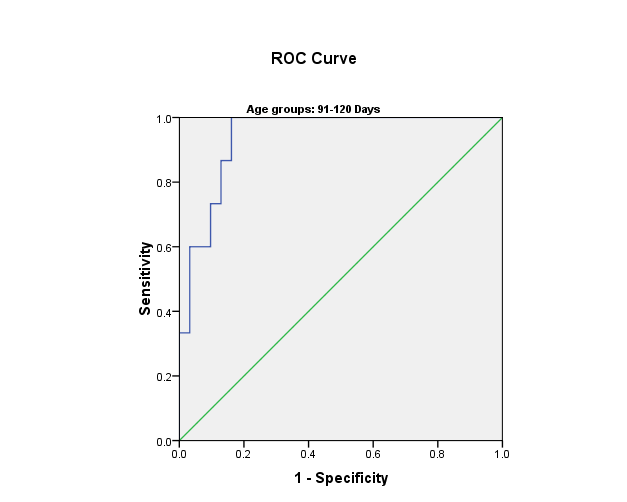  **C** |
| --- | --- | --- |
| **Figure 2.** The area under the curve (AUC) of ROC curve of GGT level in different age groups **A** AUC of 0.812 in neonate ≤ 60days (95% CI 0.754-0.871). **B** AUC of 0.868 in age group 61-90 days (95% CI 0.818-0.917). **C** AUC of 0.940 in age group from 91-120 days (95% CI 0.876-1.004). | | |
