# Supplementary material for: Pathologic approach to Neonatal cholestasis with a simple scoring system for biliary atresia
Source: Virchows Arch. 2023 Nov 27;484(1):93–102. doi: 10.1007/s00428-023-03704-5 (PMC10791702; doi:10.1007/s00428-023-03704-5)
Supplement: Supplementary file 2 — (DOCX 23 kb) [file 428_2023_3704_MOESM2_ESM.docx]

Supplementary Table 5. Histopathologic features of 162 BA cases divided according age (more or less 6 weeks).

|  | **≤ 6 weeks (N=26)** | **> 6weeks (N=136)** | ***P*-value** ^*^ |
| --- | --- | --- | --- |
| **Diagnosis Pathologically**   1. BA**^a^** (obstructive cholestasis) 2. Paucity 3. Neonatal hepatitis | 18 (69.2%)  1 (3.8%)  7 (26.9%) | 136 (100%)  0  0 | *P* < .001 |
| **Histopathologic features** | | | |
| 1. **Portal tract edema:** Present | 5 (19.2%) | 111 (81.6%) | *P* < .001 |
| 1. **Portal fibrosis**   Absent  Mild fibrosis  Moderate fibrosis  Advanced fibrosis  Cirrhosis | 1 (3.8%)  13 (50%)  11 (42.3%)  1 (3.8%)  0 | 0  19 (14%)  63 (46.3%)  53 (39%)  1 (0.7%) | *P* < .001 |
| 1. **Bile ductular proliferation**   No  Mild focal diffuse  Mild diffuse  Moderate or Marked | 1 (3.8%)  3 (11.5%)  13 (50%)  9 (34.6%) | 1 (0.7%)  2 (1.5%)  31 (22.8%)  102 (75%) | *P* < .001 |
| 1. **Ductal plate malformation**   Present | 6 (23.1%) | 33 (24.3%) | *P* = .90 |
| 1. **Portal inflammation**   Mild  Moderate | 25 (96.2%)  1 (3.8%) | 113 (83.1%)  23 (16.9%) | *P* = .09 |
| 1. **Cholangiolitis:** Present | 7 (26.9%) | 60 (44.1%) | *P* = .10 |
| 1. **Bile duct/ductular plugs:** Present | 24 (92.3%) | 135 (99.3%) | *P* = .02 |
| 1. **Cholestasis**   Mild,  Moderate  Marked | 5 (19.2%)  21 (80.8%)  0 | 11 (8.1%)  122 (89.7%)  3 (2.2%) | *P* = .17 |
| 1. **Parenchymal inflammation**   No  Mild,  Moderate | 18(69.2%)  8(30.8%)  0 | 108(79.4%)  27(19.9%)  1(0.7%) | *P* = .33 |
| 1. **Extramedullary hematopoiesis**   Present | 14 (53.8%) | 27 (19.9%) | *P* < .001 |
| 1. **Giant-cell transformation**   Present | 7 (26.9%) | 31 (22.8%) | *P* = .65 |
| 1. **Secondary siderosis:** Present | 23 (88.5%) | 33 (24.3%) | *P* < .001 |

Abbreviations: **^a^**BA (biliary atresia).

^*^ *P* < .05 is significant.
